# Supplementary material for: Different pathways for engulfment and endocytosis of liquid droplets by nanovesicles
Source: Nat Commun. 2023 Feb 4;14:615. doi: 10.1038/s41467-023-35847-z (PMC9899248; doi:10.1038/s41467-023-35847-z)
Supplement: Supplementary file 1 — Supplementary Information [file 41467_2023_35847_MOESM1_ESM.pdf]

Different pathways for engulfment and endocytosis of liquid droplets by nanovesicles

Supplementary Information

Rikhia Ghosh[1], Vahid Satarifard[1], and Reinhard Lipowsky*[1]

[1]Theory & Biosystems, Max Planck Institute of Colloids and Interfaces, 14424 Potsdam, Germany

This Supplementary Information contains the following items:

Fig. S1: Different views of the non-axisymmetric shape in Fig. 4a at $t = 30\,\mu$s.

Fig. S2: Different views of the non-axisymmetric shape in Fig. 4b at $t = 10\,\mu$s.

Fig. S3: Components of axisymmetric stress or pressure tensor.

Fig. S4: Geometry of partially engulfed droplet, defining the parameters in Eq. (12).

Fig. S5: Intrinsic contact angle θ^*_α and tilt angle $\psi_{\rm co}$ as functions of lipid number N_{ol}.

Table S1: Numerical parameter values corresponding to the data in Fig. 2.

Table S2: Numerical values of the geometric parameters that enter Eq. (12).

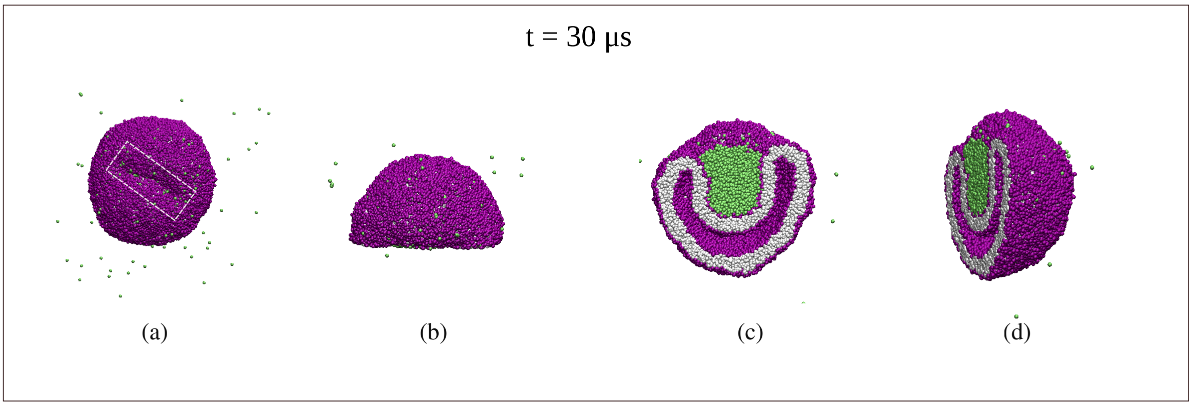

Figure S1: Different views of the non-axisymmetric vesicle-droplet morphology in Fig. 4a at $t = 30\,\mu$s: (a) Top view with a strongly non-circular contact line which is enframed by the white dashed rectangle; (b-d) Half cut of vesicle-droplet shape parallel to the long side of the white dashed rectangle. This half cut shape is shown from the top in (b), from the front in (c), and via an oblique side view in (d). The different views in (c) and (d) directly reveal the tight-lipped shape of the membrane neck. The vesicle is assembled with $N_{ol} = 5700$ lipids in the outer leaflet.

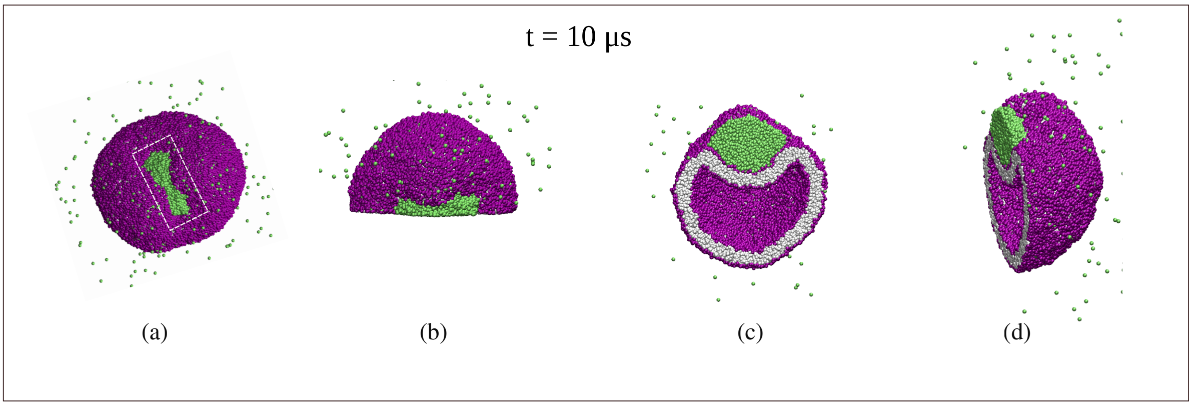

Figure S2: Different views of the non-axisymmetric vesicle-droplet morphology in Fig. 4b at $t = 10\,\mu$s: (a) Top view with a strongly non-circular contact line and $\alpha\beta$ interface (green), which are enframed by the white dashed rectangle; (b-d) Half cut of vesicle-droplet shape parallel to the long side of the white dashed rectangle. This half cut shape is shown from the top in (b), from the front in (c), and via an oblique side view in (d). The different views in (c) and (d) directly reveal the tight-lipped shape of the membrane neck. The vesicle is assembled with $N_{ol} = 5963$ lipids in the outer leaflet.

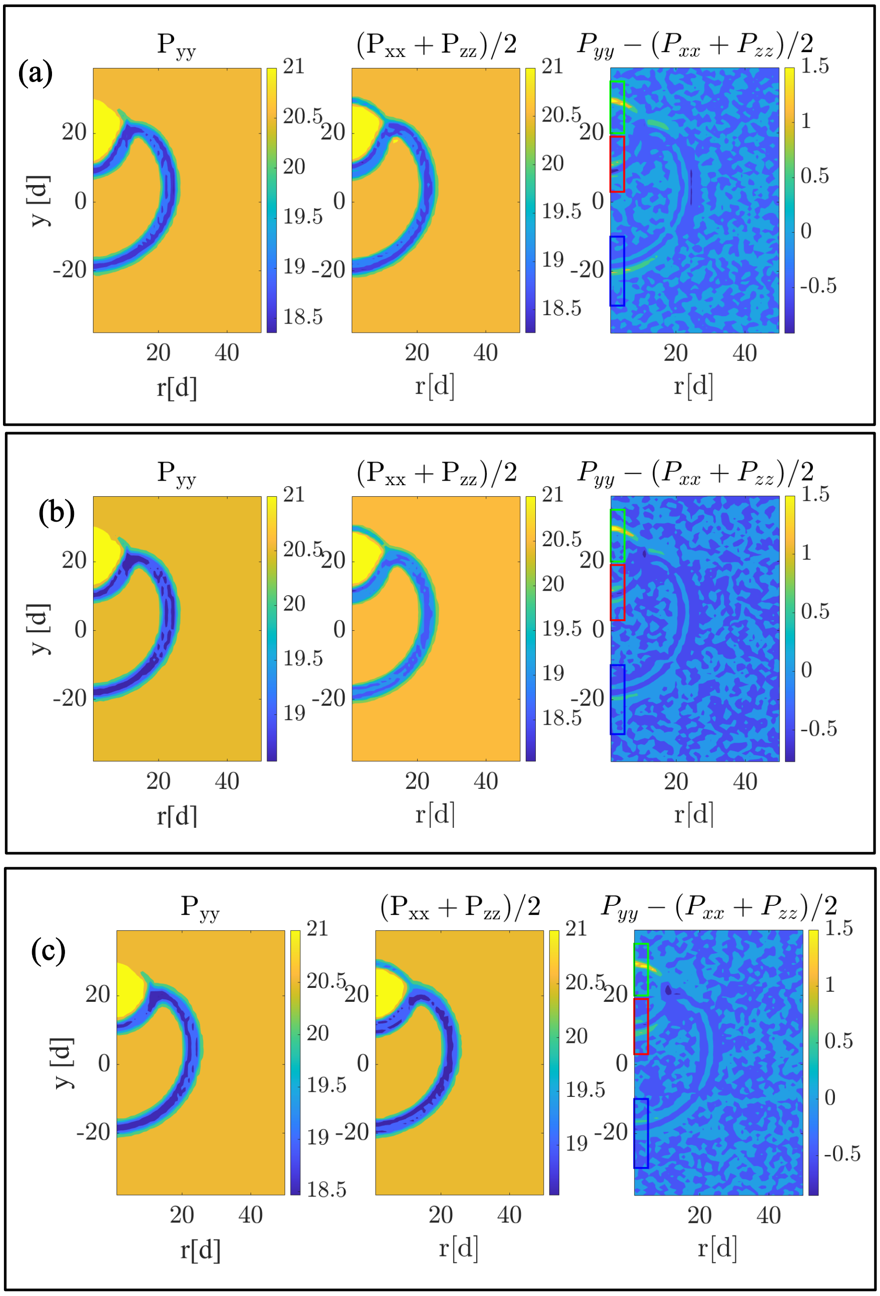

Figure S3: Axisymmetric components of the local stress or pressure tensor; vesicle-droplet systems with adjusted volume $\nu = \nu_0$, partially engulfing a droplet of intermediate diameter $18.7\,d$ or 15 nm. The three panels (a), (b), and (c) differ in their transbilayer asymmetry as determined by the lipid numbers N_{ol} and N_{il} in the outer and inner leaflet with constant total number $N_{ol} + N_{il}$ equal to 10 100. The outer leaflet is assembled from $N_{ol} = 5400$, 5700, and 5963 lipids in (a), (b), and (c), respectively. The components P_{xx}, P_{yy}, and P_{zz} of the local pressure tensor depend on the radial coordinate r and on the coordinate y parallel to the axis of rotational symmetry. In each panel from left to right, the first diagram displays the component P_{yy} of the local pressure, the second diagram the combination $\frac{1}{2}(P_{xx} + P_{zz})$, and the third diagram the stress profile $s \equiv P_{yy} - \frac{1}{2}(P_{xx} + P_{zz})$. All pressure components are given in units of $k_B T/d^2$. In all the cases, the stress profiles vary from $s = 0$ (dark blue) to $s = 1.5$ (yellow). The mechanical tension of the liquid interface $\Sigma_{\alpha\beta}$ is calculated in the green box, while the bilayer tensions $\Sigma_{\alpha\gamma}$ and $\Sigma_{\beta\gamma}$ of the membrane segments are computed in the red and blue boxes, respectively.

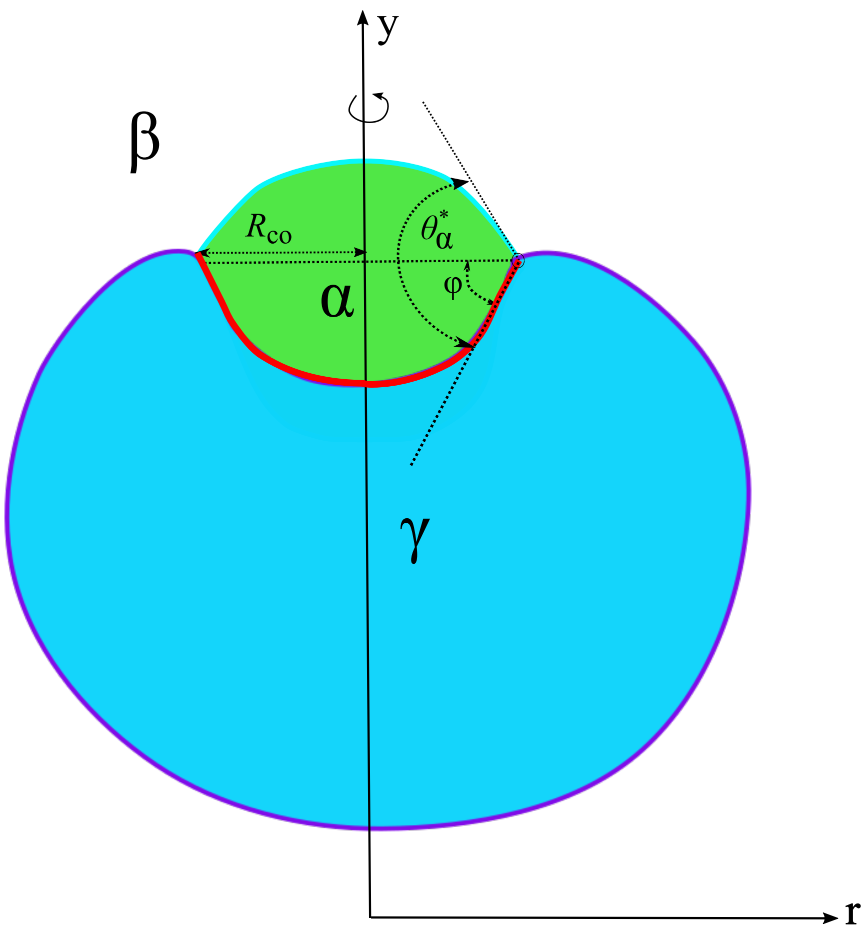

Figure S4: Schematic cross-section of a liquid droplet (green) partially engulfed by a nanovesicle filled by γ phase (blue). The coordinate along the axis of rotational symmetry is denoted by y and the radial distance from this axis is measured by the coordinate r. The contour of the membrane shape consists of two membrane segments, the $\alpha\gamma$ (red) and $\beta\gamma$ (purple) segments. The droplet is bounded by the $\alpha\gamma$ membrane segment and the $\alpha\beta$ interface (light blue) which separates the droplet from the liquid bulk phase β (white). The horizontal black line corresponds to the cross-section of the contact line. The geometric parameters that enter the force balance Eq. (12) are (i) the intrinsic contact angle θ^*_α between the membrane contour and the interface contour; (ii) the tilt angle ψ_{co} of the membrane contour at the contact line, which is equal to $\psi_{co} = -\varphi$ with φ displayed in the figure; and (iii) the contact line radius R_{co}. The negative sign of the tilt angle arises from the counter-clockwise rotation of the tangent vector at the shape contour as we move from the (indented) north pole towards the contact line. The tangent vector at the north pole is parallel to the r-coordinate and points away from the axis of rotational symmetry. The numerical values of these geometric quantities are displayed in Table S2.

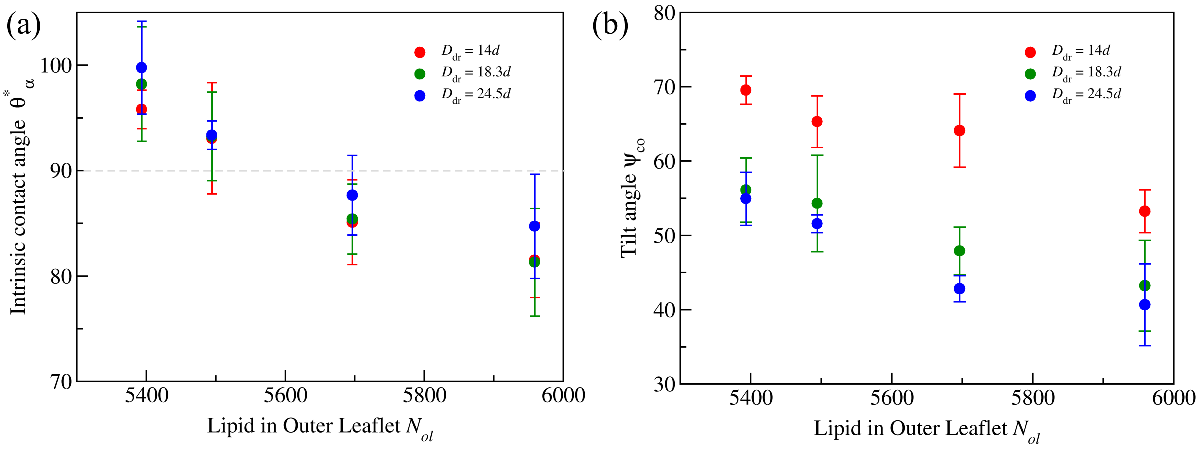

Figure S5: Partially engulfed droplets with three different diameters: (a) Intrinsic contact angle θ_α^* and (b) Tilt angle $\psi_{\rm co}$ of the membrane contour at the contact line as functions of the lipid number N_{ol}, which determines the transbilayer asymmetry. Both quantities enter the force balance Eq. (12) in the main text which is used to deduce the line tension λ. The numerical values of both θ_α^* and $\psi_{\rm co}$ are displayed in Table S2, together with the contact line radius $R_{\rm co}$. Each data point represents the mean value over $n = 15$ statistically independent samples obtained from three replicated simulations. Each error bar is the standard error of the mean (SEM).

Table S1: Numerical values for the parameters of the nanovesicles used in this study: Lipid numbers N_{ol} and N_{il} assembled in the outer and inner leaflets of the vesicle membranes; adjusted volume $\nu = \nu_0$; associated bilayer tension Σ; leaflet tensions Σ_{ol} and Σ_{il} of the outer and inner leaflets as displayed in Fig. 2 of the main text. All tensions are given in units of $k_B T/d^2$.

N_{ol}	5400	5500	5700	5963
N_{il}	4700	4600	4400	4137
ν_0	0.965	0.966	0.966	0.966
Σ	0.01	0.01	0.04	0.01
$\Sigma_{\rm il}$	-1.64 ± 0.07	-1.35 ± 0.05	-0.82 ± 0.06	-0.02 ± 0.05
$\Sigma_{\rm ol}$	1.65 ± 0.05	1.36 ± 0.04	0.86 ± 0.03	0.03 ± 0.06

Table S2: Numerical values of the geometric parameters that enter the force balance Eq. (12): Droplet diameter $D_{\rm dr}$ in units of bead diameter d and in nanometers; lipid number N_{ol} in the outer leaflet of the nanovesicle; intrinsic contact angle θ^*_α; tilt angle $\psi_{\rm co}$ of the membrane contour at the contact line; contact line radius $R_{\rm co}$ and contact line tension λ. The tilt angle $\psi_{\rm co}$ is negative for out-wetting as considered here because the tangent vector of the shape contour is rotated counterclockwise as we move from the (indented) north pole towards the contact line.

$D_{\rm dr}$ [d]	$D_{\rm dr}$ [nm]	N_{ol}	θ^*_α	$\psi_{\rm co}$	$R_{\rm co}$ [d]	λ [$k_B T/d$]
14	11.2	5400	95.8 ± 1.8	$-(69.5 \pm 1.9)$	7.2 ± 0.7	19.44 ± 4.2
		5500	93.1 ± 5.3	$-(65.3 \pm 3.5)$	7.5 ± 0.6	7.04 ± 3.1
		5700	85.1 ± 4.0	$-(64.1 \pm 4.9)$	8.2 ± 0.9	-10.03 ± 2.09
		5963	81.5 ± 3.5	$-(53.3 \pm 2.9)$	8.7 ± 0.7	-23.25 ± 5.1
18.7	15	5400	98.2 ± 5.4	$-(56.1 \pm 4.3)$	10.5 ± 0.9	17.49 ± 5.2
		5500	93.2 ± 4.2	$-(54.3 \pm 6.5)$	10.9 ± 0.8	6.17 ± 3.8
		5700	85.4 ± 3.3	$-(47.9 \pm 3.2)$	11.6 ± 1.0	-11.35 ± 4.11
		5963	81.3 ± 5.1	$-(43.2 \pm 6.1)$	11.9 ± 0.9	-26.28 ± 5.1
24.5	19.6	5400	99.8 ± 4.4	$-(54.9 \pm 3.6)$	11.9 ± 0.9	15.2 ± 4.1
		5500	93.4 ± 1.4	$-(51.6 \pm 1.2)$	12.4 ± 1.1	3.26 ± 2.4
		5700	87.7 ± 3.8	$-(42.8 \pm 1.8)$	13.1 ± 0.9	-13.61 ± 4.9
		5963	84.7 ± 4.9	$-(40.6 \pm 5.5)$	14.1 ± 1.2	-26.25 ± 5.8
