# Supplementary material for: Different pathways for engulfment and endocytosis of liquid droplets by nanovesicles
Source: Nat Commun. 2023 Feb 4;14:615. doi: 10.1038/s41467-023-35847-z (PMC9899248; doi:10.1038/s41467-023-35847-z)
Supplement: Supplementary file 3 — Description of Additional Supplementary Files [file 41467_2023_35847_MOESM3_ESM.pdf]

Different pathways for engulfment and endocytosis of liquid droplets by nanovesicles

Description of additional supplementary files

Rikhia Ghosh[1], Vahid Satarifard[1], and Reinhard Lipowsky*[1]

[1]Theory & Biosystems, Max Planck Institute of Colloids and Interfaces, 14424 Potsdam, Germany

Legends for Supplementary Movies

Movies S1 and S2: Time-lapse movies corresponding to Fig. 3a.

Movies S3 and S4: Time-lapse movies corresponding to Fig. 3b.

Movies S5 and S6: Time-lapse movies corresponding to Fig. 4a.

Movies S7 and S8: Time-lapse movies corresponding to Fig. 4b.

Legends for Supplementary Movies

Movies S1 and S2:

Endocytosis of liquid droplet (green) by nanovesicle (purple), which is formed by a lipid bilayer with $N_{ol} = 5400$ lipids in the outer and $N_{il} = 4700$ lipids in the inner leaflet as in Fig. 3a. The droplet is completely engulfed by the vesicle membrane after time $t = 0.3\,\mu$s (2nd frame). Subsequently, the membrane neck undergoes fast fission, thereby generating a small intraluminal vesicle that encloses the droplet at $t = 0.5\,\mu$s (3rd frame). The vesicle-droplet morphology remains axisymmetric during the whole process. In addition to the snapshot at $t = 0.3\,\mu$s, the two time lapse movies display 15 consecutive simulation snapshots up to $7\,\mu$s, corresponding to time increments of only $0.5\,\mu$s.

Movies S3 and S4:

Endocytosis of droplet (green) by nanovesicle (purple), which is formed by a lipid bilayer with $N_{ol} = 5500$ lipids in the outer and $N_{il} = 4600$ lipids in the inner leaflet as in Fig. 3b. Compared to the Movies S1 and S2, 100 lipids have been reshuffled from the inner to the outer leaflet, which acts to slow down the endocytic process: The droplet is now completely engulfed at time $t = 4\,\mu$s and the closed membrane neck undergoes fission at $t = 9\,\mu$s, generating a small intraluminal vesicle that encloses the droplet. The vesicle-droplet morphology remains axisymmetric during the whole process. The two time lapse movies display 61 consecutive simulation snapshots up to $30\,\mu$s, including the four snapshots in Fig. 3b. The Movie S4 demonstrates that the membrane neck undergoes fission at $9\,\mu$s and that the resulting morphology of two nested vesicles remains unchanged up to $30\,\mu$s.

Movies S5 and S6:

Complete engulfment of droplet (green) by nanovesicle (purple), which is formed by a lipid bilayer with $N_{ol} = 5700$ lipids in the outer and $N_{il} = 4400$ lipids in the inner leaflet, corresponding to the simulation snapshots in Fig. 4a. At time $t = 0$, the vesicle with volume $\nu = 0.7$ and the partially engulfed droplet have an axisymmetric shape with a circular contact line. This morphology persists until $t = 12\,\mu$s, when we reduce the vesicle volume from $\nu = 0.7$ to $\nu = 0.6$ which leads to complete engulfment of the droplet and to a non-axisymmetric morphology of the vesicle-droplet couple. The broken rotational symmetry is directly apparent from the top views in Movie S5 between $t = 12.5\,\mu$s and $t = 35\,\mu$s, which display a closed membrane neck with an elongated, tight-lipped shape, see also white dashed rectangles in Fig. 4a. The tight-lipped neck shape prevents the fission of this neck.

Movies S7 and S8:

Complete engulfment of droplet (green) by a nanovesicle (purple), which is formed by a lipid bilayer with $N_{ol} = 5963$ lipids in the outer and $N_{il} = 4137$ lipids in the inner leaflet, corresponding to the simulation snapshots in Fig. 4b. The vesicle volume is kept at the constant value $\nu = 0.7$. At time $t = 0$, the droplet is partially engulfed by the vesicle membrane with an axisymmetric contact line. This symmetry is broken at $t = 5\,\mu$s, as can be directly deduced from the non-circular contact line of the partially engulfed droplet. The droplet becomes completely engulfed at $t = 10\,\mu$s, after which the membrane forms an elongated, tight-lipped membrane neck, see top views in Movie S7 at $t = 10\,\mu$s and $t = 15\,\mu$s, see also white dashed rectangles in Fig. 4b. The tight-lipped neck shape prevents the fission of this neck.
